# Sensor Configuration and Algorithms for Power-Line Interference Suppression in Low Field Nuclear Magnetic Resonance

**DOI:** 10.3390/s19163566

**Published:** 2019-08-15

**Authors:** Xiaolei Huang, Hui Dong, Quan Tao, Mengmeng Yu, Yongqiang Li, Liangliang Rong, Hans-Joachim Krause, Andreas Offenhäusser, Xiaoming Xie

**Affiliations:** 1 State Key Laboratory of Functional Materials for Informatics, Shanghai Institute of Microsystem and Information Technology (SIMIT), Chinese Academy of Sciences (CAS), Shanghai 200050, China; 2 CAS Center for ExcelleNce in Superconducting Electronics (CENSE), Shanghai 200050, China; 3 Institute of Complex System (ICS-8), Forschungszentrum Jülich (FZJ), D-52425 Jülich, Germany; 4 Joint Research Institute on Functional Materials and Electronics, Collaboration between SIMIT and FZJ, D-52425 Jülich, Germany; 5 University of Chinese Academy of Sciences, Beijing 100049, China

**Keywords:** ultra-low field, nuclear magnetic resonance, superconducting quantum interference device, de-noising algorithms, power-line harmonics interference, J-coupling

## Abstract

Low field (LF) nuclear magnetic resonance (NMR) shows potential advantages to study pure heteronuclear J-coupling and observe the fine structure of matter. Power-line harmonics interferences and fixed-frequency noise peaks might introduce discrete noise peaks into the LF-NMR spectrum in an open environment or in a conductively shielded room, which might disturb J-coupling spectra of matter recorded at LF. In this paper, we describe a multi-channel sensor configuration of superconducting quantum interference devices, and measure the multiple peaks of the 2,2,2-trifluoroethanol J-coupling spectrum. For the case of low signal to noise ratio (SNR) < 1, we suggest two noise suppression algorithms using discrete wavelet analysis (DWA), combined with either least squares method (LSM) or gradient descent (GD). The de-noising methods are based on spatial correlation of the interferences among the superconducting sensors, and are experimentally demonstrated. The DWA-LSM algorithm shows a significant effect in the noise reduction and recovers SNR > 1 for most of the signal peaks. The DWA-GD algorithm improves the SNR further, but takes more computational time. Depending on whether the accuracy or the speed of the de-noising process is more important in LF-NMR applications, the choice of algorithm should be made.

## 1. Introduction

In the past decades, low field (LF) nuclear magnetic resonance (NMR) and magnetic resonance imaging (MRI) research has got particular attention with the goal to become a complementary supplement to conventional high-field NMR/MRI. Some potential applications of LF NMR based on both induction coil [1] and superconducting quantum interference devices (SQUID) [2,3] were investigated. LF NMR also has some successful applications, for instance, to investigate how gas hydrate accumulates and dissociates in the pore space [4]. If only low magnetic fields are needed, one can build an inexpensive and portable NMR/NMR system. As we know, the area integral under the NMR line will not change with the detection field (B_0_) strengths and signal-to-noise ratio (SNR) is defined as the ratio of the power spectral density integral under a signal spectrum to the unwanted signal spectrum [5]. When we decrease the detection field to the microtesla range, the NMR line width will be very narrow, and the peak amplitudes will increase [6]. Therefore, compared to high field, both SNR and spectral resolution will be improved in low field under the same polarization strength with high field, which permits one to study the natural NMR line width [7]. In addition, the homonuclear chemical shifts are negligible at LF, but the heteronuclear J-coupling values can be a constant before the spectrum becomes degenerate [8]. Therefore, LF NMR can be an alternative to study pure heteronuclear J-coupling [9,10], and the fine structure of matter may be observed easily. 

Larmor frequency decreases with detection field reduction. In LF, Faraday coils are unsuited for low frequency detection because their sensitivity is proportional to frequency. Therefore, the SQUID as a highly sensitive and frequency-independent magnetic flux sensor is a better choice. Both low-T_c_ and high-T_c_ SQUID sensors can be used to investigate the heteronuclear J-coupling [7,11].

Many LF NMR applications are implemented in an unshielded environment [12,13] or in a conductively shielded room made of aluminum sheets [14], with the aim to develop a portable and economic system. Under these circumstances, power-line harmonics and interferences at fixed frequencies introduce discrete noise peaks into the NMR spectrum. In order to get accurate parameters and structure estimation from the magnetic resonance spectrum, several de-noising methods have been developed. Most of them are implemented by transforming the signal and noise into another domain, such as Fourier [15], time-frequency transform [16], or wavelet transforms [17,18], and then remove the noise whose amplitudes are below a pre-defined threshold, which can be set based on the distribution of noise amplitudes [19], or be set by introducing an improved hybrid threshold function based on SNR and mean square error [20]. However, when the frequencies of the noise peaks overlap with the frequencies of the NMR signal and when the SNR is very low, the noise peaks may introduce erroneous information into the spectrum. Particularly, when people study the pure heteronuclear J-coupling at LF [21], these interferences can destroy the spectrum of the fine structure of matter. In this case, it is impossible to set an appropriate threshold. On the other hand, for de-noising the recorded signal in time domain, several effective methods have been developed to remove power-line noise from the recorded signal: notch filters [22], adaptive filters [23] and reference noise [24]. For example, notch filters were used to suppress power-line noise from NMR spectra under earth’s magnetic field [25]. However, all the three methods are only appropriate in case of a single-frequency signal. To suppress the noise in a signal bandwidth range covering hundreds of Hz, if the noise intensity is comparable with the signal intensity, an alternative de-noising method is needed.

In this work, we configure multi-channel sensors and suggest two de-noising algorithms based on the spatial correlation of the far-field interference terms in order to suppress the power-line harmonics and some interferences at fixed frequencies in SQUID-based unshielded LF-NMR spectra. We introduce reference sensors to record the power-line harmonics noise. A 2nd-order gradiometer forming the signal sensor records both NMR signal and noise at the same time. The post-processing is focused on removing the noise peaks and keeping the NMR peaks by using discrete wavelet analysis (DWA) combined with the least squares method (LSM) and the gradient descent (GD) algorithm. Using this method, we suppress the power-line harmonic interferences in time domain, and obtain clear heteronuclear J-coupling spectra of 2,2,2-trifluoroethanol at LF. This de-noising method offers the possibility to study pure J-coupling of the fine structure of matter in an unshielded or conductively shielded environment.

## 2. Materials and Methods

The following three facts are relevant in our case: (1) The NMR signal and the power-line harmonics interferences are uncorrelated because the NMR signals are transient and decay with time, but the interferences are almost continuous periodic signals. This can help us process the interferences independently, without being affected by the NMR signal. (2) In our case, the homogeneous magnetic field noise components dominate the magnetic field noise [26] and we focus on suppressing these noise components. (3) The interferences from all detectors should be strongly spatially correlated.

### 2.1. Sensor Configuration and Measurement Sequence

**Figure 1:**
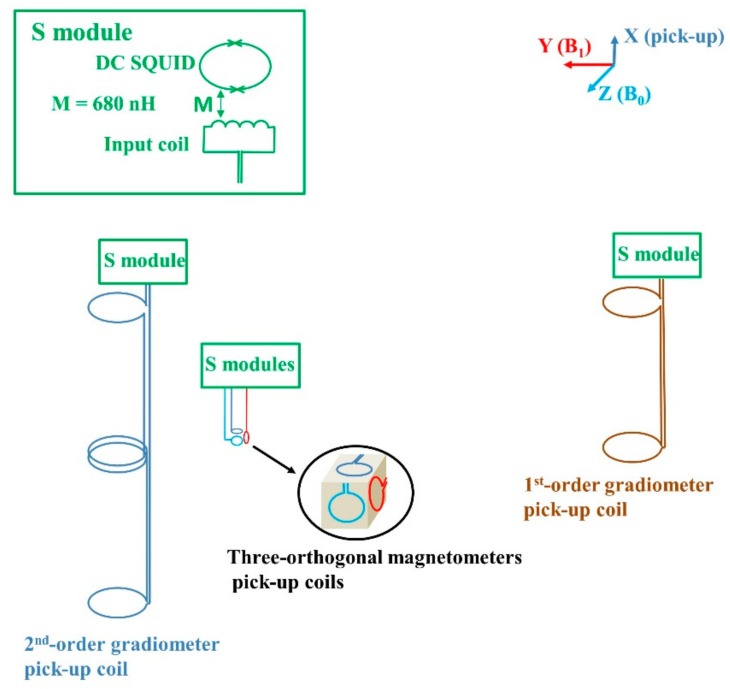
The superconducting quantum interference devices (SQUID) sensors for signal and noise recording. The ring with two crosses represents the DC SQUID with two Josephson junctions.

We configure a 4-channel SQUID sensors system which is placed in a commercial liquid helium dewar. The schematic diagram of the system can be found in [26]. The signal sensor, as shown in Figure 1, is composed of a 2nd-order gradiometer connecting to SQUID module (so-called S module in Figure 1) with 22 mm diameter and 50 mm baseline. 2nd-order gradiometers which consist of two 1st-order gradiometers connected with opposite wire-winding direction, are usually used in unshielded or conductively shielded environment to suppress both homogeneous field noise and 1st-order gradient noise. Our S module consist of an input coil and a DC SQUID with 680 nH mutual inductance. The distance between the room temperature sample and the lowest coil of the 2nd-order gradiometer is 15 mm. The reference sensors consist of three orthogonal magnetometers with 2 mm diameter connecting to S modules. They are placed about 60 mm above the sample to make sure that the amplitude of the sample NMR signal picked up by the magnetometers is below 12 fT/√Hz, which is the value of our measured environmental white noise above 3 kHz. Note that the reference sensors can also include 1st-order gradiometers in the case of strong environmental gradients. 

**Figure A1:**
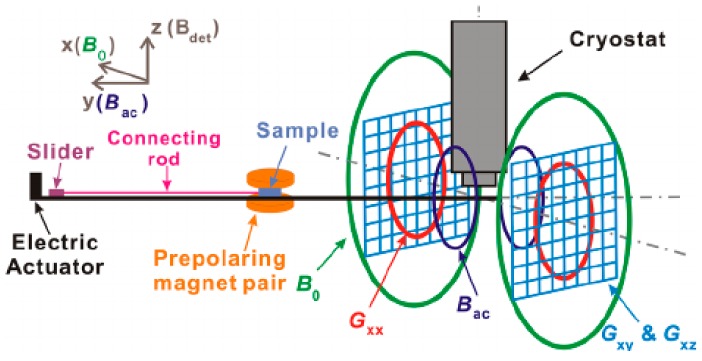
Schematic of the LF MRI system.

Our LF-NMR experiments are performed in an unshielded environment. The sample is 2,2,2-trifluoroethanol with NMR grade 99.8%. The 2,2,2-trifluoroethanol is placed in a 20 mL polyvinyl chloride bottle. A 0.65 T permanent magnet pair is employed to supply the prepolarization (B_p_) field. A motorized transportation device is used to transfer the sample from the polarizing field to the measurement location underneath the SQUID sensors. A more detailed description of the system (see Figure A1 in Appendix A) can be found in [27].

**Figure 2:**
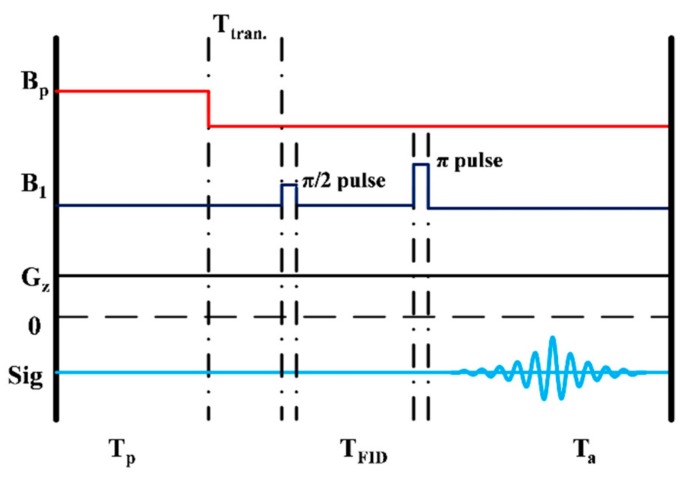
Sequence for spin echo measurement.

The experiment starts by prepolarizing the sample in the permanent magnet pair for a time T_p_ = 5 s, as shown in Figure 2. After prepolarization, the sample is transported to the outer bottom of the dewar in a transportation time of T_tran_ = 550 ms. In order to get the NMR signal at the Larmor frequency *f*_L_ of 5 kHz (B_0_ =117 μT), a π/2 and a π excitation field pulse are applied as excitation (B_1_) fields. T_FID_ is the time when the free induction decay (FID) signal relaxes completely. We record the signal with the frequency encoding gradient G_z_ switched on to bring down the SNR to a low level on purpose, e.g., to obtain an SNR of about one, in order to study the efficiency of our de-noising approach. The SQUID readout electronics is kept in the reset state during 2 ms of delay time after finishing the π pulse. Subsequently, spin echoes are recorded for a duration of T_a_.

### 2.2. Interference Suppression Algorithms 

#### 2.2.1. Discrete Wavelet Analysis-Least Squares Method (DWA-LSM) Based De-Noising

Wavelets are mathematical functions that cut up data into different levels in time domain. Each level is matched with a frequency range [28]. Although there are many applications of wavelets in NMR signal noise reduction, the appropriate de-noising technique is specific for one particular spectrum and cannot easily be generalized. Two important factors affect the DWA-based de-noising quality. The first one is the selection of the wavelet functions. The Daubechies (db) wavelet, coif wavelet and sym wavelet are generally suitable for retaining the signal. In order to limit the complexity of computation, the db wavelet is chosen [29]. The db4 wavelet attains the smallest entropy, which means the most critical information of the signal will be retained [20]. The db3 also attains a small entropy, and it can smoothen the spectrum, sharpen multi-peak shapes and at the same time maintain the peaks’ locations [30]. In addition, no signal peak will be eliminated, and weak peaks that are embedded in the noise will be recovered. So we choose db3 wavelet for interference reduction. The second key factor is the determination of a decomposition level. White noise estimation is often used to determine the decomposition level for signal with white noise [20]. Here, both the wanted signal (spin echo) and unwanted signal (power-line interference) can be seen as ‘signal’ whose features should be retained during DWA, since this is very important for the further de-noising processes. Although in our case the noise is not dominated by white noise, we want to ensure that white noise can be sufficiently weakened at each level, which helps us to focus on suppressing the power-line interferences. The optimal decomposition level is 5 for a white noise estimation based method [29]. But if the duration time of the spin echo signal is short, for example for the case of short spin-spin (T_2_) relaxation time, the frequency resolution of the signal will be limited by the duration time. In that case, the Gabor wavelets will be a better choice because they provide the best compromise between simultaneous time and frequency signal representations [31].

By using DWA to decompose the recorded spin echo signal B_echo_ and the three-axis interference components B_x,y,z_ (a simplified representation of B_x_, B_y_, B_z_) into different levels in time domain, we can process the different harmonic interferences independently. We assume that the signal frequency range is from f_1_ to f_2_, which depends on the Larmor frequency, the J-coupling strength, and the signal levels, which cover the frequency range from f_1_ to f_2_, are the levels ranging from n_1_ to n_2_. The de-noising process will be only implemented within the levels from n_1_ to n_2_, which helps us speed up the process. Now the decomposed B_echo_ at the i^th^ level can be described as:

(1) $$B_{\mathrm{echo}}{}^{i}\left(j\right)={\;S}^{i}\left(j\right)+N_{2G}{}^{i}\left(j\right),\;j\in \left[1,100000\right],\;i\in \left[n_{1},n_{2}\right],$$

where $S^{i}\left(j\right)$ is the pure echo signal at the i^th^ level, $N_{2G}{}^{i}\left(j\right)$ the noise at the i^th^ level picked up by the 2nd-order gradiometer, and j the number of data points ranging from 1 to 100000. The sampling rate of the data acquisition is 100 kHz and the data recording time T_a_ is 1 s. The key goal is to remove $N_{2G}{}^{i}\left(j\right)$ from $B_{\mathrm{echo}}\left(j\right)$ at each level. As mentioned, S(j) and $N_{2G}\left(j\right)$ are uncorrelated, because S(j) is transient and $N_{2G}\left(j\right)$ is a continuously periodic signal dominated by the homogeneous magnetic field noise from x, y and z directions. So when the echo signal ends, the B_echo_ in the i^th^ level can be described as:

(2) $$B_{\mathrm{echo}}{}^{i}\left(j\right)={\;N}_{2G}{}^{i}\left(j\right),\;j\in \left[m+1,100000\right],\;i\in \left[n_{1},n_{2}\right].$$

Here, we assume that the echo signal ends at the data point m. Now the noise suppression coefficients $k_{x,y,z}^{i}$ at the i^th^ level can be obtained by fitting the $N_{2G}{}^{i}\left(j\right)$ and the $B_{x,y,z}{}^{i}\left(j\right)$ based on the LSM with the data points ranging from m+1 to 100000, as given below:

(3) $$\sum_{j=m+1}^{100000}{\left[N_{2G}{}^{i}\left(j\right)-k_{x,y,z}^{i}\cdot B_{x,y,z}{}^{i}\left(j\right)\right]}^{2}\to \min.$$

Finally, we use $k_{x,y,z}$ to suppress the noise at each level and sum up the de-noised signals of each level. The total de-noised output $B_{\mathrm{out}}\left(j\right)$ can be written as:

(4) $$B_{\mathrm{out}}\left(j\right)=\sum_{i=n_{1}}^{n_{2}}[B_{\mathrm{echo}}{}^{i}\left(j\right)-k_{x,y,z}^{i}\cdot B_{x,y,z}{}^{i}\left(j\right)]\;j\in \left[1,100000\right].$$

A detailed description of the noise reduction process based on the DWA-LSM can be found in [26]. The effect of the LSM-based noise suppression may be limited by two reasons: (1) some near-field interference could not be suppressed by the method based on spatial correlation, (2) the least squares method finds the globally optimal solution, so the suppression coefficients matrix k can be easily dominated by the interferences with strong intensity, but the weak interferences will be ignored. However, the process of the LSM-based noise suppression method is simple and therefore suited for online de-noising.

#### 2.2.2. Discrete Wavelet Analysis - Gradient Descending (DWA-GD) Based De-Noising

We suggest the GD algorithm after 1D DWA to determine the suppression coefficients matrix $k_{x,y,z}$ for further suppression of the weak interferences. Before determining $k_{x,y,z}$, we propose normalization for data pre-processing, before applying the process of Equation (4). Normalization is particularly useful for the algorithm involving neural networks and clustering, and it helps prevent attributes with initially large range from outweighing attributes with initially smaller range [32]. We choose the min-max normalization because it can preserve the relationships among the original data values [33], and the relative amplitudes and the arrangements of the data will be retained. The formulation of the min-max normalization can be described as:

(5) $$X^{\prime }=\frac{X-X_{min}}{X_{max}-X_{min}},$$

where *X* is the original data, $X^{\prime }$ the new value linearly transformed from *X*, and $X_{max}$ and $X_{min}$ the maximal and minimal values in *X*. It can be easily seen that when $X=X_{min}$, $X^{\prime }=0$, and when *X* = $X_{max}$, $X^{\prime }$ = 1. The entire range of *X* values from $X_{min}$ and $X_{max}$ is mapped to the range from 0 to 1. The advantage is that the weight of each data point is the same. 

After the normalization of the recorded spin echo data and the three orthogonal interference components in each level, the $k_{x,y,z}$ can then be fitted based on the GD optimization algorithm. Note that the determination of k can be regarded as a linear regression model because the suppression is focused on suppressing the homogeneous magnetic field noise. In statistics, linear regression is a linear approach to modeling the relationship between a dependent variable and one or more independent variables (three orthogonal interference components B_x,y,z_). By setting *α*, *β*, *γ* as the independent variables and $\hat{Y}$ as the dependent variable, a linear relationship between these variables in each level can be described as:

(6) $$\hat{Y}=a\alpha +b\beta +c\gamma +d,$$

where $\hat{Y}$ is the predicted value for given values of *α*, *β* and *γ*. *a*, *b* and *c* are the slopes (the suppression coefficients in our case), and *d* is the intercept. Now the challenge is to determine the values of *a*, *b*, *c*, and *d*. The GD algorithm starts by introducing the mean squared error function ∆, known as the loss function, to calculate the difference between the actual *Y* (the recorded spin echo signal) and the predicted $\hat{Y}$ value [34,35]:

(7) $$\Delta \;=\;\frac{1}{2p}\sum_{i=1}^{p}{\left(Y_{i}-{\hat{Y}}_{i}\right)}^{2}=\;\frac{1}{2p}\sum_{i=1}^{p}{\left(Y_{i}-\left(a\alpha_{i}+b\beta_{i}+c\gamma_{i}+d\right)\right)}^{2},$$

where i is the index of the data point. Then we calculate the partial derivatives of the loss function with respect to *a*, *b*, *c*, and *d*, and plug in the values of *α*, *β*, *γ*, and *Y*:

(8) $$\left[\begin{array}{c} \begin{array}{c} \frac{\partial }{\partial a}\\ \frac{\partial }{\partial b}\\ \frac{\partial }{\partial c} \end{array}\\ \frac{\partial }{\partial d} \end{array}\right]\Delta =-\frac{1}{p}\sum_{i=1}^{p}\left(Y_{i}-{\hat{Y}}_{i}\right)\left[\begin{array}{c} \alpha_{i}\\ \beta_{i}\\ \begin{array}{c} \gamma_{i}\\ 1 \end{array} \end{array}\right].$$

The starting points of a_0_, b_0_, c_0_, and d_0_ can be zero or random numbers. Now we update the current values of a_c_, b_c_, c_c_, and d_c_ using the partial derivatives and the step length *L*:

(9) $$\left[\begin{array}{c} a\\ b\\ \begin{array}{c} c\\ d \end{array} \end{array}\right]=\left[\begin{array}{c} a_{c}\\ b_{c}\\ \begin{array}{c} c_{c}\\ d_{c} \end{array} \end{array}\right]-L\times \left[\begin{array}{c} \begin{array}{c} \frac{\partial }{\partial a}\\ \frac{\partial }{\partial b}\\ \frac{\partial }{\partial c} \end{array}\\ \frac{\partial }{\partial d} \end{array}\right]\Delta .$$

In the first step, the step length L_1_ can be chosen a little bit larger to speed up convergence. We repeat this process from Equations (7) to (9) until the loss function is smaller than a given threshold ε_1_, then we get the first approximation of values a_1_, b_1_, c_1_, d_1_. The threshold ε_1_ should not be too small at the first time, which can also speed up convergence. Now the first approximation of values a_1_, b_1_, c_1_, and d_1_ are close to the optimal values, but still need to be improved. Note that the results are very sensitive to the choice of the starting points a_0_, b_0_, c_0_, and d_0_, and setting them to zero or to random numbers is not the best option. In order to solve this problem, we subsequently use the first approximation values of a_1_, b_1_, c_1_, and d_1_ to be the starting points, and repeat the process again. It can be regarded as the introduction of a feedback to improve the starting points. In this round, the step length L_2_ and the threshold ε_2_ should be smaller to obtain precise results. The optimal suppression coefficients can be obtained. Then we repeat the process in Equation (4) to remove the noise from B_echo_ by using the optimal suppression coefficients.

### 2.3. Numerical Simulation

**Figure 3:**
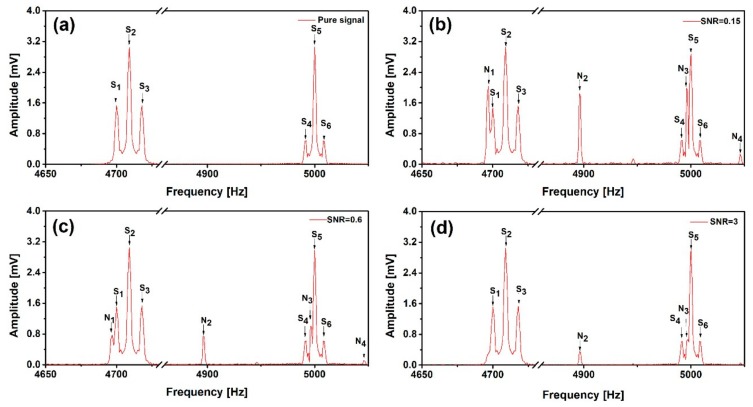
Simulated spin echo signal without noise (**a**) and with measured noise added, for different signal to noise ratio (SNR): (**b**) SNR = 0.15, (**c**) SNR = 0.6 and (**d**) SNR = 3.

We firstly verified the effectiveness of the two noise suppression algorithms using a simulated spin echo signal with two different groups, as shown in Figure 3a, whose strength distributions are 1:2:1 (S_1–3_), and 1:5:1 (S_4–6_), respectively. The duration of the simulated signal is 0.45 s and the data acquisition time is 1 s. To simulate the practical noise performance, we added some real noise picked up by the reference sensor, which contains both white noise and power-line interferences, with different noise strengths to achieve different SNR. The noise-added signals are depicted in Figure 3b, c and d, with SNR = 0.15, 0.6 and 3, respectively. Using these artificial signals covered with real noise, we can verify the de-noising effectiveness in case of low SNR. We use the ratio of the energy spectral density integral under the smallest signal peak (S_4_) and one of the strongest harmonic peaks (N_2_) which doesn’t overlap with the signal peaks, for determination of the SNR.

**Figure 4:**
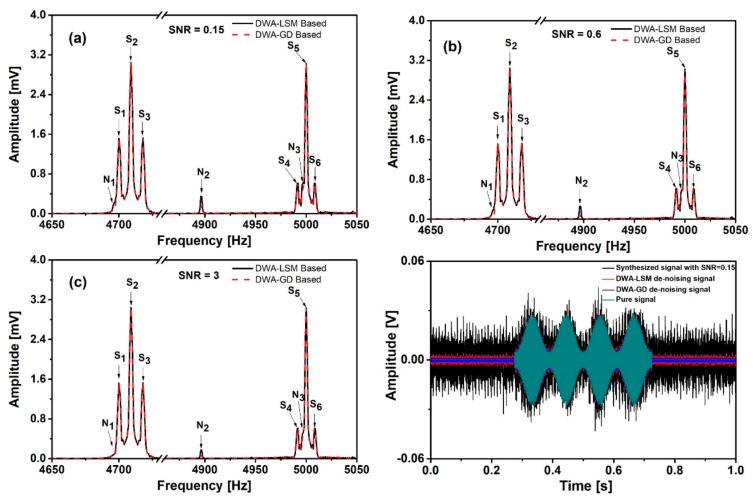
De-noised signal spectra under different SNR of (**a**) SNR = 0.15, (**b**) SNR = 0.6 and (**c**) SNR = 3. (**d**) The signals with SNR = 0.15 before and after de-noising, and the pure signal in time domain.

The noise of the synthesized signals was suppressed based on the two algorithms described in Section 2.2. Figure 4a–c show the noise suppression effects for different values of SNR. We can see that the noise peaks are greatly suppressed. In the case of lowest SNR, N_1–3_ have been suppressed by a factor of 88%, 83% and 85%, respectively, by using DWA-LSM. By using DWA-GD, N_1–3_ have been suppressed by a factor of 95%, 90% and 90%, respectively. The suppression factors can be calculated by two steps: (1) Calculate the residual noise defined as the difference between de-noised signal and pure signal. (2) Calculate the suppression factors by using the residual noise spectrum and pure noise spectrum. Figure 4d shows the signals with SNR = 0.15 before and after de-noising and the pure signal in time domain. It clearly illustrates the noise reduction in time domain. Table 1 shows the SNR changes during the de-noising process for different values of SNR. In all cases, the SNR has been significantly improved. Compared with LSM, GD yields further noise suppression. However, the GD consists of several steps and the process takes 10 min in case of SNR = 0.15, and several minutes in the other two cases (10,0000 data points). This is because the initial value of the added noise is different when we use the same step length to converge to the optimal point. A larger initial value will even take more time. For comparison, the LSM process can be finished within ten seconds. The number of data points also influences the computing time, based on the Bachmann-Landau notation [36]. When the data points double, the computing complexity will be 4 times longer than before since both the GD and the LSM involve square operations. Furthermore, the result is very sensitive to the step length set in the GD. If the step size is too large, the output might not converge to the optimal solution. If the step is too small, the convergence will be very slow. The SNR is limited by the residual noise whose amplitude is about 5% of the original noise amplitude in Figure 3b. The accuracy of the loss function and the step length are the main reasons that limit further noise reduction. However, higher accuracy and smaller step length will take more time and memory. In practical application, if the amplitudes of the noise peaks are significantly smaller than the amplitudes of the signal peaks, comparable to the white noise, a lower accuracy is acceptable.

Note that before the suppression but after addition of the noise, the amplitudes of S_1_ and S_5_ reduce in Figure 3b–d. The lower the SNR, the larger the reduction. After the suppression, the amplitudes of S_1_ and S_5_ recover from the reduction. For example, compared to the pure signal, the amplitudes of S_1_ and S_5_ reduce by a factor of 4.5% and 5.9% in Figure 3b, and recover to 0.7% and 1% in Figure 4a. It is clear that the frequencies of S_1_ and S_5_ are very close to the noise peaks, so that their amplitudes can be affected when we suppress the noise in the time domain. The amplitude can be increased or decreased, depending on the phases of signal and noise. It can be explained that in the extreme case, when two signals with the same frequency but different phases pass through a linear system at the same time, the amplitude and phase of the output signal become a combination of these two signals. When one of the two signals is removed, the amplitude and phase of the remaining one will be recovered from the combination. Since S_2,3,4,6_ are stable and S_1,5_ change no more than 1%, it can be concluded that 99% NMR information has been preserved.

## 3. Results and Discussion

### 3.1. Spectral Correlation Coefficients

**Figure 5:**
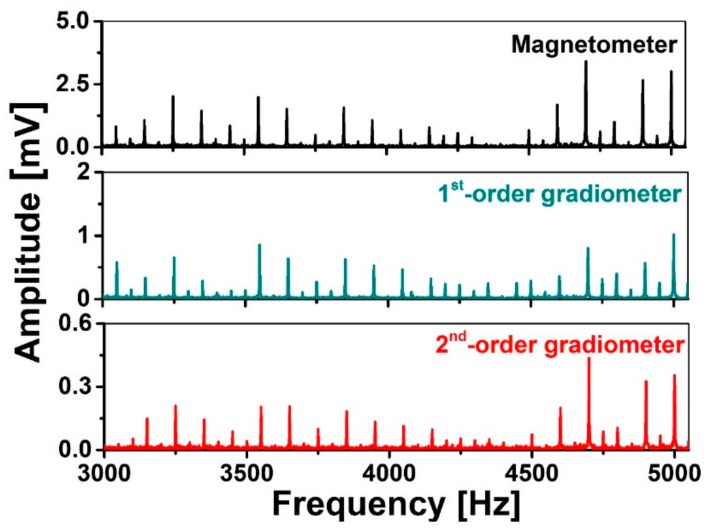
The environmental noise spectra measured by a magnetometer with 2 mm diameter, 1st- and 2nd-order gradiometers with 22 mm diameter and 50 mm baselines. All pick up coils are wound using 80 μm diameter Nb wire.

Firstly, we study the spectral correlation among the noise spectra of the different sensors, the magnetometer, the 1st- and 2nd-order gradiometers, between 3 and 5 kHz, as shown in Figure 5. The results show that there are two harmonics every 100 Hz, so if the signal bandwidth or J-coupling bandwidth is more than 50 Hz, the interference will overlap with the signal spectrum. The noise spectral correlation coefficient among the detectors can then be calculated by using the Pearson correlation coefficient (PCC), as shown in Table 2. The PCC is a measure of the strength of the association between two variables [37]. The number between −1 and 1 indicates the extent to which two variables are linearly related. The values of 1 and −1 imply complete positive and negative correlations, respectively. A value of 0 implies that the two variables are uncorrelated. We found correlation coefficients above 0.89, and the correlation coefficient between the magnetometers and the second-order gradiometer reached a value of 0.97. This means that the frequencies of the harmonics picked up by different sensors are the same, and the relative output amplitudes of the harmonics from each sensor are similar. Although the values of the correlation coefficients will change with the measurement position, all the three coefficients at the three measurement positions amount to more than 0.80. In order to achieve a good spectral correlation coefficient, the measurement system should be located far away from near-field sources (e.g., neighboring electronic devices).

### 3.2. Interference Suppression Based on the DWA-LSM

**Figure 6:**
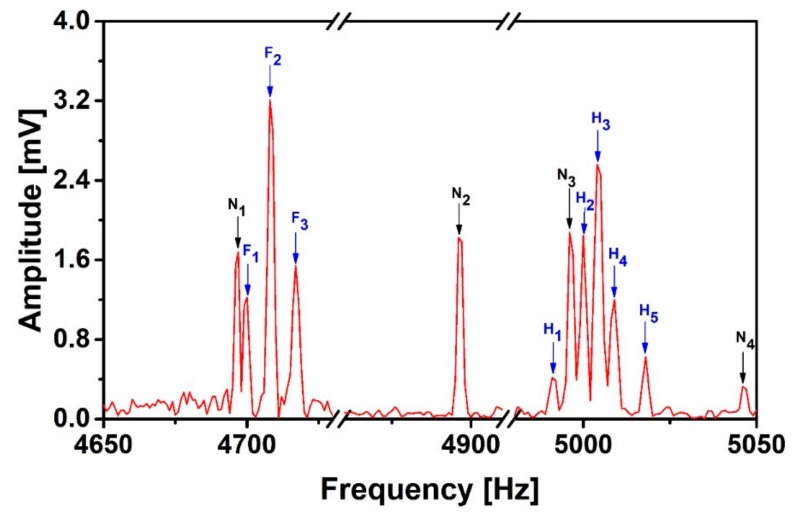
J-coupling spectrum of 2,2,2-trifluoroethanol (signal shot). N_1,2,3,4_ are the noise interference peaks, F_1,2,3_ the fluorine peaks, and H_1,2,3,4,5_ the proton peaks.

Based on the good spectral correlation between the 2nd-order gradiometer and the magnetometer, we choose the 2nd-order gradiometer as the signal sensor to record spin echo signals of 20 mL 2,2,2-trifluoroethanol at 5 kHz proton Larmor frequency. The power-line harmonics interferences are recorded by three orthogonal magnetometers at the same time. In order to verify our de-noising method at low SNR, we implement a 2 µT/m frequency encoding gradient during the measurement to reduce the SNR. Figure 6 shows the heteronuclear J-coupling spectra of the 2,2,2-trifluoroethanol NMR signal and the 50 Hz harmonics interferences between 4650 and 5050 Hz which are picked up by the signal sensor. The fluorine and the proton groups of peaks are well separated, with center frequencies of about 4708 Hz and 5004 Hz, respectively. The linear broadband detector SQUID can easily record the signal groups from two different nuclei. We can clearly observe 3 fluorine peaks (F_1_–F_3_) and 5 proton peaks (H_1_–H_5_). As expected, the strength distribution of 1:2:1 for triplet fluorine peaks due to the coupling of the three fluorine nuclei with the two protons can be easily observed. The OH is uncoupled, so the proton spectrum appears as five proton peaks. The proton-fluorine coupling strength is about 9 Hz, which is in good agreement with measurement results at high-field [38] and in the earth’s magnetic field [39]. The line width of the NMR spectra is 1 Hz, which is limited by the data acquisition time 1 s and by the frequency encoding gradient. However, the interferences (N_1_–N_3_) destroy the spectrum of the fine structure of matter and make it impossible to get accurate parameters and structure estimation from the NMR spectrum. We also use the ratio of the energy spectral density integral under each signal peak and N_2_ for determination of SNR. For most of the signal peaks, the SNR is much less than 1.

**Figure 7:**
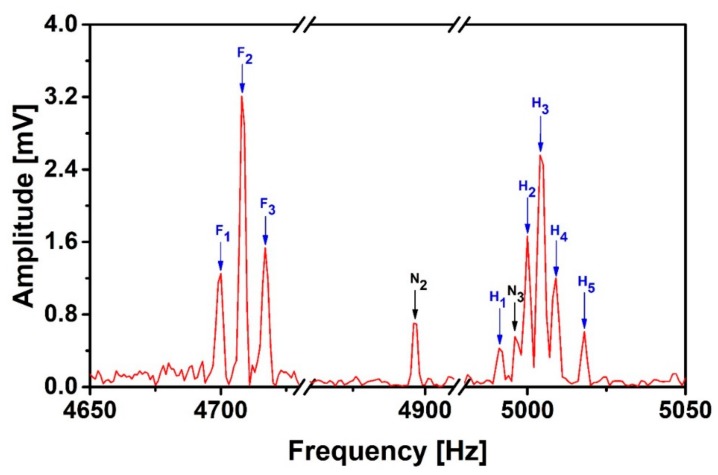
J-coupling spectrum of 2,2,2-trifluoroethanol after applying the Discrete Wavelet Analysis-Least Squares Method (DWA-LSM) suppression method.

For noise suppression in the case of SNR < 1 and noise distribution covering the signal frequency range (e.g., N_1_, N_3_), we firstly decompose the B_echo_ and the B_x,y,z_ using 1D DWA and suppress the noise based on the LSM. Figure 7 shows the suppression effect. The noise peaks N_1_ and N_4_ were removed, the amplitudes of N_2_ and N_3_ were significantly suppressed by a factor of 2.6 and 3.4, respectively. We assume the noise peaks are removed when their amplitudes are below the white noise. Both the frequencies and the amplitudes of most of the fluorine and proton peaks are almost unchanged. However, some noise peaks are still present and their amplitudes are comparable with the weakest proton peak H_1_. The energy spectral density integral under N_2_ has been suppressed by a factor of 6.5. For most of the signal peaks, the SNR has become more than 1, but the integrals under F_1_ and H_2_ have changed by 6.7% and −6%, respectively. The reasons can be explained from two aspects: (1) as mentioned in 2.3, the signal amplitudes can be affected by nearby noise peaks. (2) The fitting error due to nonlinear items in the interference may disturb the signals close to the noise peaks. In general, the interference peaks need to be further suppressed.

### 3.3. Optimizing the Determination of the Suppression Coefficients

**Figure 8:**
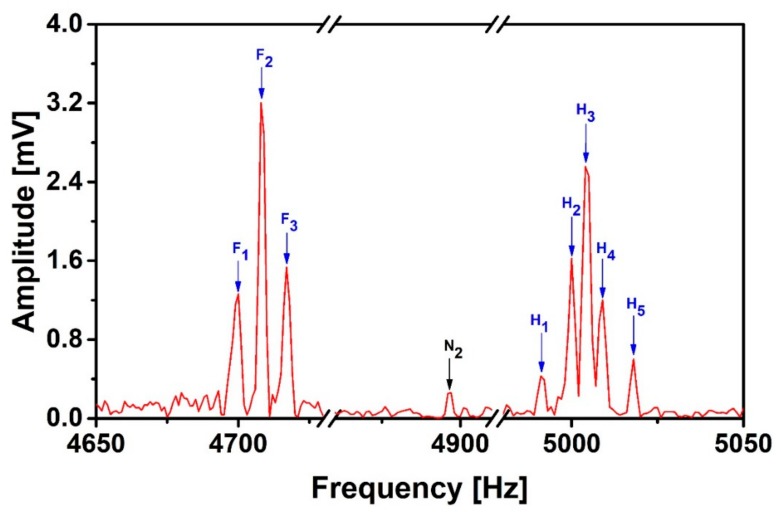
J-coupling spectrum of 2,2,2-trifluoroethanol after applying the Discrete Wavelet Analysis-Gradient Descent (DWA-GD) suppression method.

In order to further improve the SNR, we implement normalization for data pre-processing and suppress the interferences based on the DWA-GD. As shown in Figure 8, all the interference peaks are further suppressed as compared to Figure 7, and the amplitude of N_2_ has been reduced by a factor of 2.8 and N_3_ has been removed. The weakest proton peak H_1_ is larger than all interference peaks. Table 3 lists the calculated SNR and the energy spectral density integrals under the signal and noise peaks. The integral under N_2_ has been suppressed by a factor of 7.3 when using DWA-GD de-noising process compared to that with DWA-LSM. With DWA-GD, we improve the SNR of all signal peaks to be larger than 1. The integrals under F_1_ and H_2_ become a bit larger, but the total change is no more than 10%, which is much less than the change of the area under N_2_. The rest of the fluorine and proton peaks are retained well. Compared to the LSM, the GD can achieve a better noise reduction effect and a wider signal frequency bandwidth, and the min-max normalization ensures that the data points are not overwhelmed by each other. However, compared with the numerical simulation in 2.3, the GD takes 20–30 min, even more than the 10 min in the simulation, and the noise peaks amplitude suppression factors reduce from 95% in the simulation to 87% in the practical application. This is because the noise recorded at the signal sensor is slightly different than that at the reference sensor, but is the same in the simulation since the noise in the synthesized signal is taken from the reference sensor. The different white noise in the signal and reference sensors makes the noise distribution more complicated than in the synthesized signal, which takes more computing time and reduces the suppression factor. For the case that SNR is not too low, people can choose the LSM only to speed up the process with an acceptable noise suppression effect. 

## 4. Conclusions

To study pure heteronuclear J-coupling in ultra-low field in an unshielded environment with power-line harmonics interference, we suggested two noise suppression methods based on the DWA-LSM and the DWA-GD optimal algorithms. We firstly verified the effectiveness of two de-noising methods on reducing the noise in the synthesized signal. We then demonstrated the DWA-LSM suppression procedure using spectral data recorded from a 2,2,2-trifluoroethanol sample. The result showed that the SNR had been significantly improved, while the signal peaks which were close to the interference peaks changed no more than 7%. In order to further suppress the interferences, we developed a second method to determine the suppression coefficients based on the DWA-GD. Before determining the suppression coefficients, we used the min-max normalization to ensure that every data point had the same weight. The result illustrated that the amplitude of the noise peak N_2_ was suppressed by a factor of 7.28 and the SNR was improved by a factor of 47 if we ignored the change in the energy spectral density integral under F_1_ and H_2_ which was no more than 10%. With suitable choice of starting point and step length, both algorithms show good robustness in case of low SNR. Depending on whether accuracy or speed of the de-noising process is more important in LF-NMR applications, the choice of algorithm should be made. The algorithm should work in the case of multi-peaks J-coupling spectra with low SNR at ultra-low field, and also for MRI in the unshielded or conductively shielded case.

## Figures and Tables

**Table 1:** Signal to noise ratio (SNR) before and after the de-noising process by using the Discrete Wavelet Analysis-Least Squares Method (DWA-LSM) and Discrete Wavelet Analysis-Gradient Descent (DWA-GD).

**SNR before De-Noising**			0.15	0.6	3
**SNR**	After DWA-LSM		2.9	5.3	11
	After DWA-GD		12.5	36	45
**The factors of SNR improvement**	After DWA-LSM		19.3	8.8	3.6
	After DWA-GD		83.3	60	15

**Table 2:** The Pearson correlation coefficients among the three sensors calculated from Figure 5.

PCC	Magnetometer	1st-Order Gradiometer	2nd-Order Gradiometer
**Magnetometer**	1	0.91624	0.97076
**1st-Order Gradiometer**	0.91624	1	0.89323
**2nd-Order Gradiometer**	0.97076	0.89323	1

**Table 3:** The calculated energy spectral density integral under the signal and noise peaks, as well as the SNR before and after the de-noising process by using the DWA-LSM and DWA-GD.

**Recorded Signal and Noise Peaks**			F_1_	F_2_	F_3_	H_1_	H_2_	H_3_	H_4_	H_5_	N_2_
Energy spectral density integral under peaks (10^−6^ J) *	Before de-noising		3.3	22	5.56	0.38	5.11	15.3	3.09	0.6	6.62
	After DWA-LSM		3.52	22	5.53	0.39	4.8	15.3	3.08	0.58	1.02
	After DWA-GD		3.62	22	5.53	0.4	4.5	15.3	3.08	0.57	0.14
**SNR**	Before de-noising		0.5	3.32	0.83	0.06	0.77	2.3	0.46	0.09	/
	After DWA-LSM		3.45	21.56	5.42	0.38	4.71	15	3.02	0.57	
	After DWA-GD		25.85	157.1	39.5	2.85	32.1	109.3	22	4.07	

* We use energy spectral density because NMR signal is a transient signal which only has spectral energy.
